# Delayed Reproduction, Injury, and Regeneration of Testes in Out-of-Season Breeding of Largemouth Bass (*Micropterus nigricans*)

**DOI:** 10.3390/antiox13091077

**Published:** 2024-09-04

**Authors:** Kuo He, Yi Yang, Zhihong Li, Haoxiao Yan, Kaige Song, Qiao Liu, Liulan Zhao, Song Yang

**Affiliations:** College of Animal Science and Technology, Sichuan Agricultural University, Chengdu 611130, China; hk101010@yeah.net (K.H.); 13547857587@163.com (Y.Y.); 15826317295@163.com (Z.L.); yhx07272022@hotmail.com (H.Y.); 14986@sicau.edu.cn (K.S.); liuqiaosau@163.com (Q.L.)

**Keywords:** *Micropterus nigricans*, testis damage, low-temperature stress, oxidative stress, hormones, apoptosis

## Abstract

Out-of-season breeding is an effective method for addressing seasonal shortages of fry in aquaculture species such as largemouth bass (LMB) for year-round production. Off-season breeding of LMB can be achieved by subjecting breeding LMB to prolonged low-temperature conditions; however, this can alter reproductive rhythms, affecting the quality of their sperm and leading to a decrease in reproductive efficiency. Therefore, it is crucial to investigate issues such as the damage to the testes and the related mechanisms caused by low-temperature stress during out-of-season breeding. In this experiment, we assessed the changes in the testes during this time in LMB by comparing reproductive rhythms, testicular histomorphology, ultrastructure, antioxidant capacity and apoptosis. We synthesized measurements of LMB from three identically treated cement ponds and fish exposed to water temperatures of 13–16 °C to assess the changes in the testes. The results showed that (1) out-of-season reproduction delayed sperm production and promoted sperm redevelopment in LMB, various hormone levels have changed over time (e.g., LH, FSH, and T). (2) The head plasma membrane of LMB spermatozoa was separated, and the middle mitochondria were swollen. (3) The expression levels of antioxidant enzymes (cat, sod, and gpx) were upregulated, and oxidative stress occurred in LMB. (4) The expression levels of apoptosis genes (e.g., *bax*, *bcl2*, and *caspase3*) were upregulated, and apoptosis occurred in LMB due to off-season breeding. Moreover, important genes of the mitochondrial apoptosis pathway (bid, CYT-C) were upregulated, indicating that spermatozoan apoptosis in LMB was probably achieved through the mitochondrial apoptosis pathway. These results suggest the delays, damage, and regeneration of LMB testes. Our findings provide new insights into the molecular mechanisms that trigger changes in sperm quality during out-of-season breeding in fish.

## 1. Introduction

Studies of fish reproductive behavior and efficiency have shown that most fish have a fixed breeding season that leads to seasonal shortages of fish fry, resulting in a significant increase in the demand for off-season fry [1]. Out-of-season breeding of largemouth bass can be realized through artificial intervention, thereby realizing higher economic efficiency [2]. Water temperature is one of the most important factors in achieving out-of-season breeding. Some studies have shown that largemouth bass and semi-smooth-tongue sole (*Cynoglossus semilaevis*) parents, when transferred to appropriate water temperature conditions, can realize off-season breeding [3,4]. The currently achievable out-of-season breeding does not compare favorably with natural reproduction, and most studies that have examined the causal factors have focused on egg quality. Therefore, investigating the changes in spermatozoa during off-season breeding may be key to improving and optimizing such breeding techniques.

Water temperature is an important abiotic factor that controls reproduction in most fish species by influencing gamete maturation [5]. Out-of-season breeding of largemouth bass can be achieved by programmed cooling, but abnormal changes in water temperature can cause severe stress to fish and adversely affect the testis [6]. Intense hypothermic stress leads to changes in sperm structure, causing histological damage to the head and middle of the spermatozoa [7]. In addition, the hypothalamus, after receiving costimulatory signals, influences the concentration of relevant hormones (i.e., luteinizing hormone [LH] and follicle-stimulating hormone [FSH]) through the hypothalamus–pituitary–gonadal axis (HPG) that ultimately influences the testicular maturation process [8,9]. It has been reported that hypothermic stress leads to the production of ROS in the testes, and the excess ROS lead to oxidative stress that impairs the physiological functioning of the testes [10]. Prolonged hypothermia increases the expression of genes involved in spermatogonia apoptosis, which ultimately triggers spermatozoan apoptosis [11]. Sustained hypothermic stimulation is an important factor in gonadal redevelopment. Therefore, there are multiple processes of change in the testes during out-of-season reproduction, which can affect testicular development and sperm quality. However, the extent of the effects and the specific mechanisms need to be further investigated.

Out-of-season breeding of LMB is a method of reproduction that increases economic efficiency. However, this method may affect reproductive rhythms, damage gametes, and lead to low reproductive rates. Therefore, understanding the mechanisms that affect it is key to achieving efficient out-of-season breeding [12]. Current research on out-of-season breeding has generally focused on the ovary, but the testes play an equally important role in reproduction, and we have found that a lot of changes happen to the fish testicles during this process. Therefore, the purpose of this experiment was to study the changes and damages to the testis during out-of-season breeding, and to understand the mechanisms and causes of such damages. The findings of the study will provide a theoretical basis for the subsequent improvement in reproduction efficiency in out-of-season breeding of largemouth bass.

## 2. Materials and Methods

### 2.1. Experimental Protocol

Approximately 6000 LMB (initial mean weight 285.3 ± 70.0 g) that had reached sexual maturity for the first time were procured from a farm in Qionglai City, Sichuan Province, China. The fish were equally distributed among three identical concrete ponds (17 × 3.5 m; water depth 1.2 m), with 2000 fish in each pond. The fish were cultured in natural water from a spring near the farm, and the water temperature was maintained between 13 and 16 °C (see Supplementary Figure S1 for the monthly average water temperature). The experimental period lasted for 153 days (3.20–8.26). Every day at 17:00, the fish were provided with Tongwei commercial feed (Chengdu, China), which contains a minimum of 46% crude protein and 5% crude fat. During the experimental period, the dissolved oxygen in the water was maintained at or above 5.6 mg/L, and the levels of ammonia and nitrite were kept below 0.04 mg/L.

### 2.2. Tissue Sampling

The sampling dates for this experiment were March 20, April 24, May 26, June 27, July 28, and 26 August 2022. For each sampling, nine male LMB were randomly collected from each cement pond, anesthetized with MS-222 (0.1 mg/mL, Sigma, Dalian, China), and the body weight, carcass weight, gonad weight, and liver weight were measured. The gonadosomatic index (GSI) and hepatosomatic index (HSI) were calculated. Venous blood collection was performed from the tail of the anesthetized fish using a 5 mL syringe. The blood was allowed to clot completely and then centrifuged for 20 min at 3000 rpm, and the supernatant was used as serum. The testes of the fish were collected, and a portion of the testes was frozen in liquid nitrogen and stored at −80 °C for gene expression and enzyme expression assays. Some portions of the testes were stored at room temperature for histological analysis and electron microscopic observation.

### 2.3. HE Staining

At each sampling time, three fish testes from each pool were randomly selected for histopathological examination. The samples were initially fixed in 4% paraformaldehyde to preserve their structural integrity. After fixation, they underwent a graded ethanol dehydration process to remove excess water. The samples were then immersed in xylene to aid embedding, followed by embedding in paraffin. Then, the paraffin-embedded samples were sectioned into 5 µm slices using a room-temperature microtome (Olympus BX43, Tokyo, Japan) and a cryostat (CM1950, Leica Biosystems, Wetzlar, Germany). The sections were initially stained with hematoxylin, followed by differentiation and bluing. Subsequent staining was performed with eosin. The sections were then subjected to a dehydration process through sequential immersions in ascending concentrations of alcohol and xylene. Following dehydration, the sections were mounted in neutral resin and examined under a microscope (Nikon, Eclipse, Ti-S, Tokyo, Japan) after thorough drying.

### 2.4. Transmission Electron Microscopy

Initially, the samples were prefixed in 3% glutaraldehyde for approximately 24 h, followed by postfixation in 1% osmium tetroxide for 2 h. The next step involved sequential dehydration of the testicular tissues through graded acetone solutions (30%, 50%, 70%, 80%, 95%, and 100%), with each concentration being applied for 20 min, and 100% acetone was used twice for 15 min each. Subsequently, embedding was performed using a series of acetone and Epon812 resin mixtures in the ratios of 3:1, 1:1, and 1:3, with the final embedding in pure Epon812 resin. Ultrathin sections, approximately 60–90 nm in thickness, were prepared with an ultramicrotome, collected on 200-mesh copper grids previously stained with uranyl acetate (10–15 min, protected from light), and further stained with lead citrate (1–10 min) at room temperature. The grids were then washed three times in ultrapure water, gently blotted with filter paper, and left to air-dry overnight in a copper grid box at room temperature. Imaging and analysis were subsequently conducted using a JEM-1400FLASH transmission electron microscope (JEOL, Tokyo, Japan).

### 2.5. Serum Hormone Determination

Serum hormone levels were determined using an enzyme-linked immunosorbent assay (ELISA) kit from Shanghai Enzyme Linkage Biotechnology (Shanghai, China) [13]. Luteinizing hormone (LH), follicle stimulating hormone (FSH), testosterone (T), and estradiol (E2) were experimentally manipulated in fish according to the instructions of the kit, and solid-phase antibodies were prepared by coating microtiter plates with purified capture antibodies against LH, FSH, T, and E2. Finally, the absorbance values were measured at 450 nm using a spectrophotometer, and the levels of LH, FSH, T, and E2 in serum were calculated using standard curves.

### 2.6. mRNA Expression Analysis

Total RNA was extracted from the sperm nest tissues according to the instructions of an animal total RNA isolation kit (Foregene, Chengdu, China) and stored at −80 °C. cDNA was constructed using PrimeScript^TM^ RT reagent using a gDNA Eraser Kit (Foregene, Chengdu, China). Quantitative real-time polymerase chain reaction (qRT-PCR) was performed on a CFX Connect™ Real-time PCR system (Bio Rad, USA). RT-qPCR analysis of the mRNA was performed using EASYTm-SYbr Green I (Cat. No. Qp-01011, Foregene). The qPCR temperature profile for all genes was 95 °C for 20 s, 40 thermal cycling steps consisting of 3 s at 95 °C, 30 s at TM, and 72 °C for 60 s. Primers for the relevant qPCR analysis were designed according to previous studies by our group [3] and are listed in Table S1. Template amounts were normalized using β-actin and 18 s as internal reference genes. The relative transcript levels of the genes were calculated using the 2^−ΔΔCt^ method.

### 2.7. Western Blot

Testis tissues were lysed with 500 μL of protein lysate and centrifuged for 15 min to extract the supernatant. The target proteins were transferred to a precast gel for electrophoresis and then transfected onto a PVDF membrane and incubated with bovine serum protein for two hours. Excess primary antibody was removed with TBST, and then the membrane was transferred to secondary antibody culture solution and incubated for one hour, after which the excess antibody was removed by washing with TBST. Finally, a color development solution was added to the PVDF membrane to react with the target protein, and the protein bands were detected by the experimental apparatus. Protein detection results were calculated using Image J software (version 1.5.4). The antibodies used were CASPASE3 Rabbit Antibody (1:500, YT0668, Immunoway, Plano, TX, USA), CAT Rabbit Antibody (1:1000, A11220, Immunoway), and β-actin Mouse Antibody (1:1000, 200068-8F10, Zenbio, Durham, NC, USA).

### 2.8. Statistical Analysis

Data (mean ± SEM) were organized using Excel and analyzed by SPSS statistical software (SPSS Inc.; Chicago, IL, USA, version 22.0). One-way analysis of variance (ANOVA) and multiple comparisons (Duncan’s multiple range test) were used to statistically analyze the results of gene expression and serum hormone levels in the sperms of largemouth bass every month. Different letters in the figure indicated significant differences. GraphPad Prism 8.0 (GraphPad Software Inc., San Diego, CA, USA) was used for graphing.

## 3. Results

### 3.1. Average Weight, GSI, and HSI

**Figure 1:**
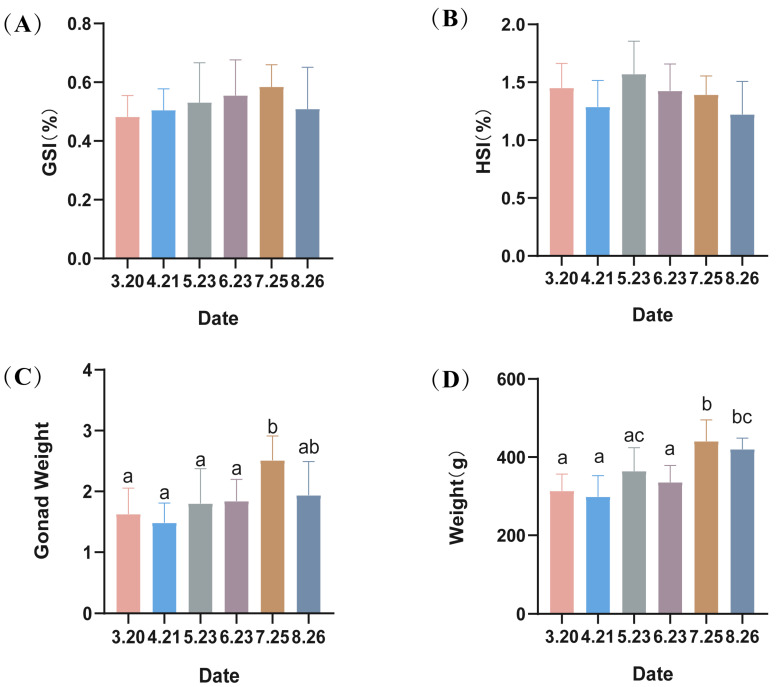
(**A**–**D**) Hepatosomatic index (HSI), gonadosomatic index (GSI) and the average weight of largemouth bass. Different lowercase letters indicate significant differences in the same group at different times (*p* < 0.05).

There were no significant differences (*p* > 0.05) in the gonadal index (GSI) or liver index (HSI) of male LMB (Figure 1). However, there were significant increases in gonad weight and body weight of LMB in July.

### 3.2. Histopathological Evaluations

**Figure 2:**
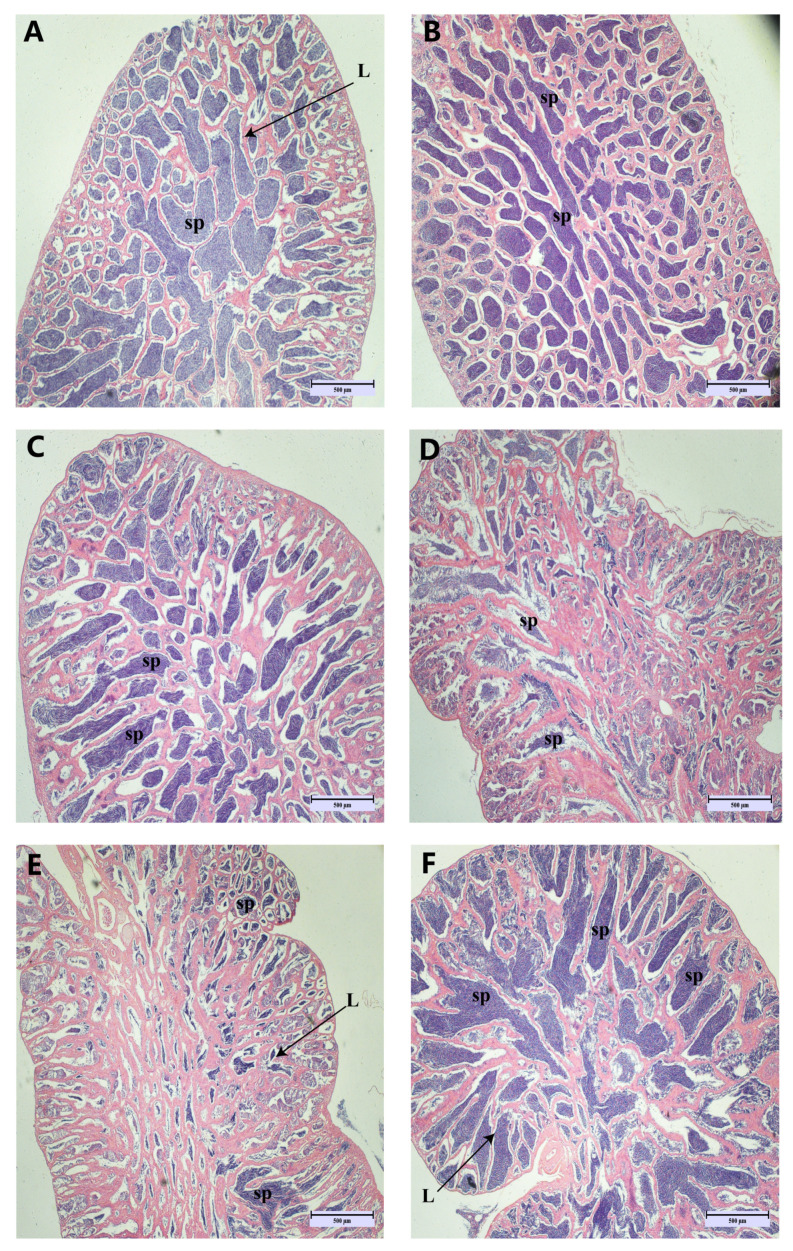
Histopathological appearance of spermatogonial tissue. (Magnification ×40, scale bar: 500 μm). Images (**A**–**F**) correspond to the histological section maps of March, April, May, June, July, and August. sp: spermatozoa; L: lobular lumen.

In the early stages of out-of-season reproduction, the testes are already in a state of maturity (Figure 2A,B). However, spermathecae underwent sperm discharge as well as sperm re-maturation during May–August (Figure 2C–F), and newly produced spermatogonia were observed in June and July (Figure 2D,E). The observation of the testes of LMB revealed that most of the testes were in stage IV, and only those in June were in stage III (Supplementary Figure S2).

### 3.3. Electron Microscopic Examination

**Figure 3:**
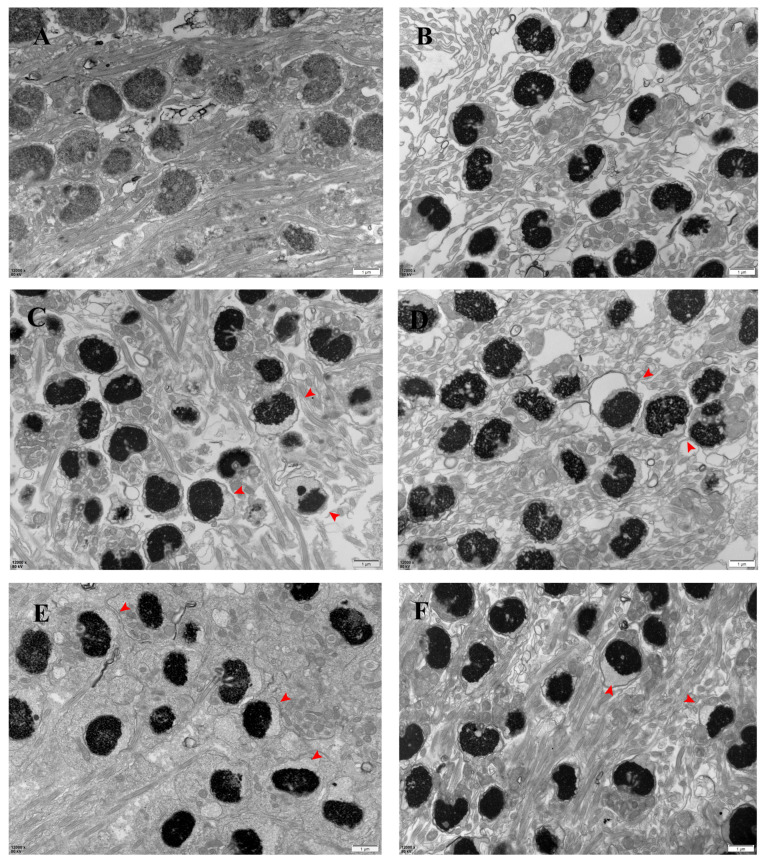
Electron micrographs of the largemouth bass’s spermary (scale bar: 1 μm). Note: images (**A**–**F**) correspond to March, April, May, June, July, and August samples. n: nucleus; red arrow: head plasma membrane separation.

The ultrastructure of the spermatozoa was normal in March (Figure 3A); separation of the plasma membrane gradually began to appear in the head of the spermatozoa in April (red arrow in the figure), and the separation of the plasma membrane of the spermatozoa gradually increased with time (Figure 3B–F). The ultrastructure of the sperm tail was normal from March to August, and the “9 + 2” structure was maintained.

**Figure 4:**
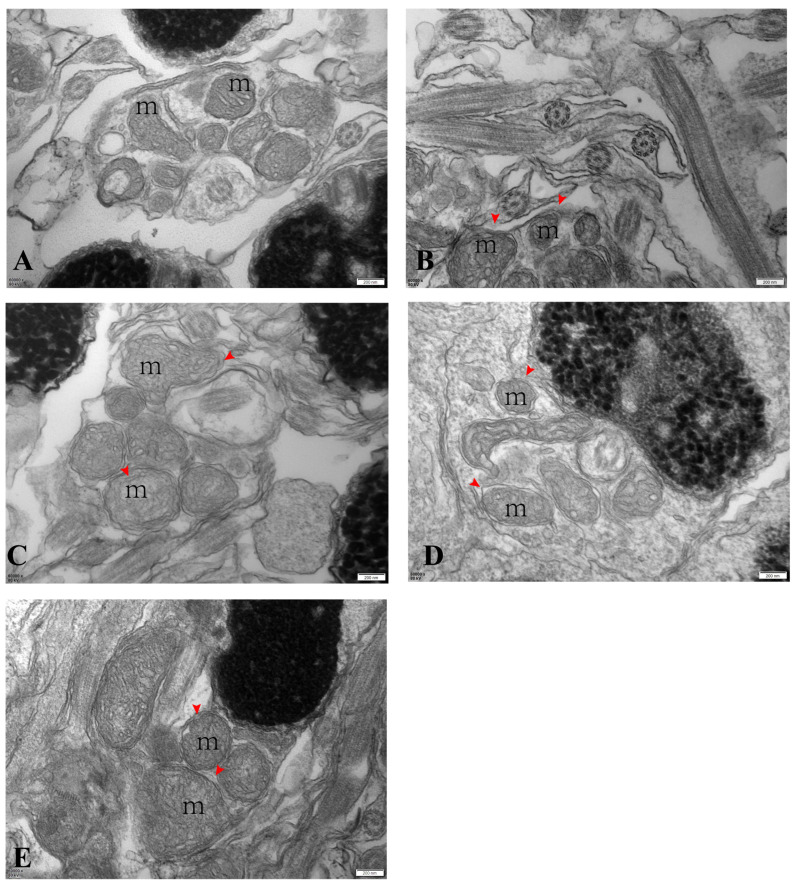
Electron micrographs of largemouth bass’s spermary (scale bar: 200 nm). Note: images (**A**–**E**) correspond to samples from April, May, June, July, and August. m: mitochondria; red arrow: swollen mitochondria in the center.

The ultrastructure analysis showed that most of the mitochondrial structures were abnormal starting from the introduction of the low-temperature stress in April. The middle segments of spermatozoa from April to August showed apparent mitochondrial swelling, disappearance of mitochondrial cristae, and unclear boundaries of mitochondrial membranes (Figure 4A–E).

Observation of the ultrastructure of the spermatozoa of LMB in May showed plasma membrane separation in the sperm heads and mitochondrial swelling in the middle segments of the sperm (Supplementary Figure S3).

### 3.4. Expression Levels of Membrane-Related Genes

**Figure 5:**
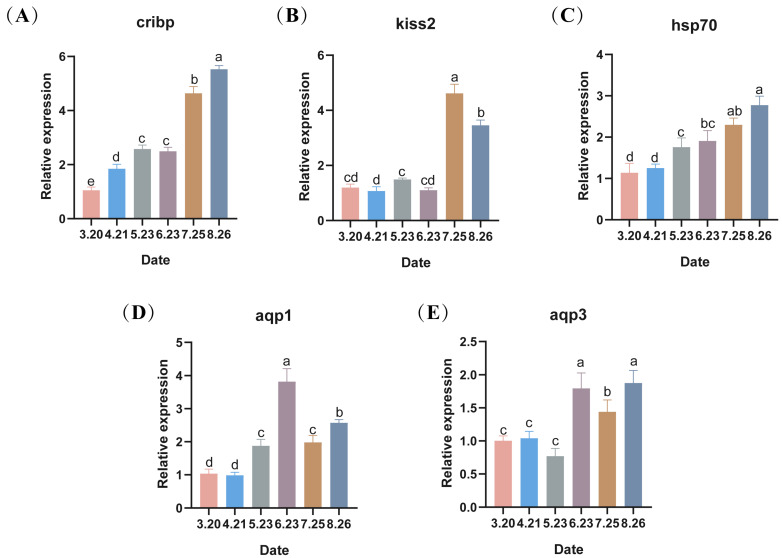
(**A**–**E**) The changes in the expression levels of low-temperature marker genes and repair protein-related genes during the autumn breeding of largemouth bass, with different lowercase letters indicating significant differences at different sampling time points (*p* < 0.05).

With the onset of low-temperature stress in LMB during out-of-season culture, the expression of the low-temperature marker gene *cirbp* in the testes gradually increased except in May and June, showing significant differences between months (*p* < 0.05), with its highest value in the final month (Figure 5A). Kisspeptin 2 (*kiss2*) was significantly expressed in July as well as in August (*p* < 0.05) (Figure 5B). Moreover, heat shock protein 70 (*hsp70*), a repair gene, showed an increasing trend, reaching its maximum in August (*p* < 0.05) (Figure 5C). Aquaporin 1 (*aqp1*) expression showed an increasing trend, followed by a decreasing trend, reaching its maximum level in June (Figure 5D). Aquaporin 3 (*aqp3*) expression was similar, also highly expressed in June, but its maximum level was reached in August (*p* < 0.05) (Figure 5E).

### 3.5. Determination of Hormone-Related Indices

**Figure 6:**
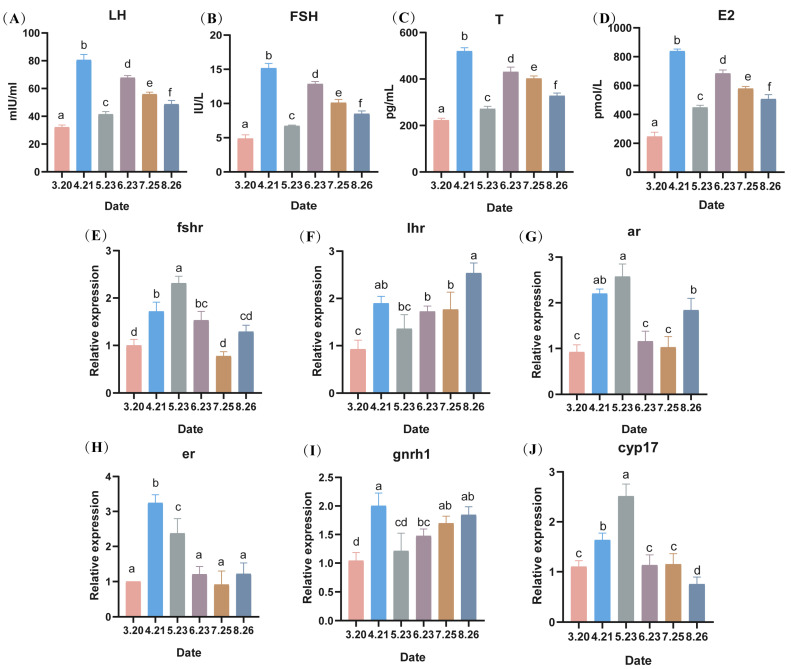
Effect of serum hormone levels, expression levels of hormone-related receptors and steroid hormone synthesis-related genes in largemouth bass (**A**–**D**): changes in hormone levels, (**E**–**J**) expression levels of hormone-related receptors in sperm and their steroid hormone synthesis-related genes) during counter-seasonal breeding. Note: Data are expressed as mean ± SD (*n* = 9). Different lowercase letters indicate significant differences at different sampling time points (*p* < 0.05).

The serum hormones of LMB (LH, FSH, T, and E2) showed a surge in expression in April, a decrease in expression in May, and a gradual decrease from June to August, with a significant difference between months (*p* < 0.05). The expression levels of hormones in April–August were higher than those in March (i.e., without the low-temperature stress) (Figure 6A–D). Similar to the hormonal results, all hormone receptors showed an increasing trend in the first month of cold stress, and the expression levels of follicle-stimulating hormone receptor (*fshr*), estrogen receptor (*er1*), 17 family, subfamily A, and polypeptide 1 (*cyp17a1*) were lower in June, July, and August (Figure 6E,H,J). Luteinizing hormone receptor (*lhr*) as well as gonadotropin-releasing hormone 1 receptor (*gnrh1*) showed an increasing trend in transcript levels in June, July, and August (Figure 6F,I).

### 3.6. Oxidative Stress Parameters Assays

**Figure 7:**
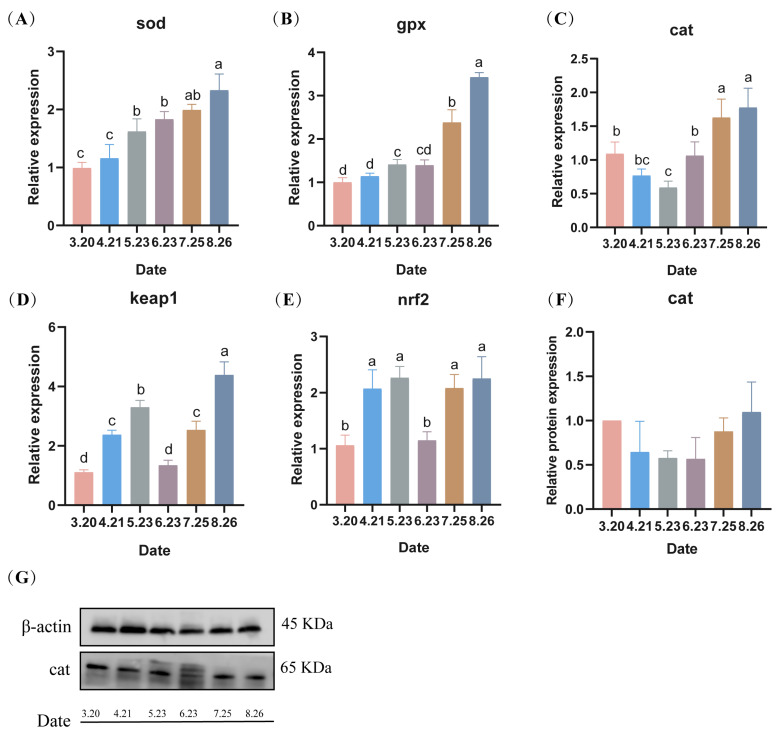
(**A**–**G**) Effect of low temperature on the expression levels of genes and proteins related to antioxidant enzymes in spermatozoa during fall reproduction. Note: Data are expressed as mean ± SD (*n* = 9). Different lowercase letters indicate significant differences between controls at different sampling time points (*p* < 0.05).

Under low-temperature stress, the expression of *sod* genes in LMB testes gradually increased with the prolongation of stress, reaching a maximum in August (Figure 7A). Similar to Superoxide dismutase (*sod*), Glutathione peroxidase (*gpx*) in LMB testes showed a gradual increase during fall reproduction, reaching a maximum in August (*p* < 0.05) (Figure 7B). Catalase (*cat*) genes of LMB sperm showed a trend of decreasing and then increasing under low temperature, reaching a minimum value in May (*p* < 0.05) and then increasing, reaching a maximum value in August (*p* < 0.05) (Figure 7C). Moreover, the expression of the antioxidant-related gene *keap1* (kelch-like ECH-associated protein 1) of LMB showed an overall increasing trend with the prolongation of low-temperature stress (Figure 7D). In addition, the expression of the antioxidant-related gene *nrf2* (Nuclear factor erythroid 2) also showed a significant increase with the prolongation of low-temperature stress (Figure 7E). The protein expression levels of the LMB testes *cat* during fall breeding were similar to the gene expression levels, both showing a decreasing trend, followed by an increasing trend (Figure 7F,G).

### 3.7. Measurement of Apoptosis-Related Indices

**Figure 8:**
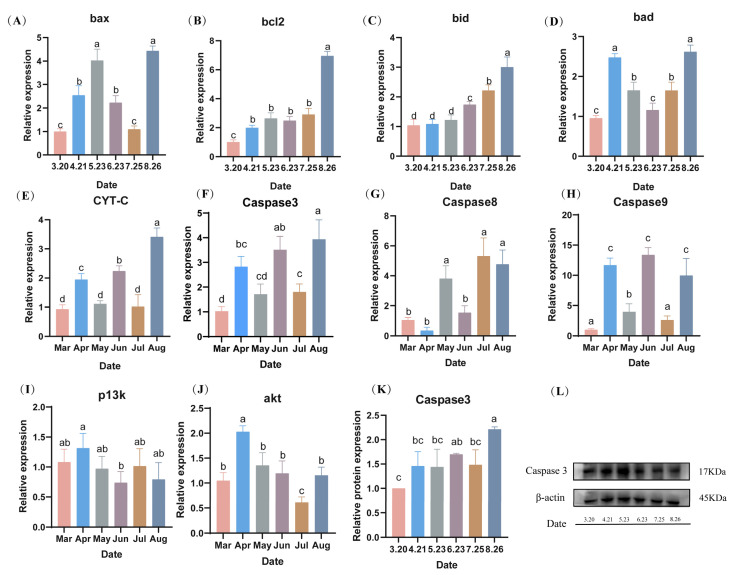
(**A**–**L**) Effect of hypothermia on the expression levels of apoptosis-related genes and proteins in the spermathecae during fall reproduction. Note: Data are expressed as mean ± SD (*n* = 9). Different lowercase letters indicate significant differences from controls at different sampling time points (*p* < 0.05).

During the fall breeding of LMB, the mRNA expression levels of sperm nest pro-apoptotic factors, including B-cell lymphoma-2 (*bcl2*), BCL2-Associated X (*bax*), BCL-2 interacting death protein (*bid*), BCL2-associated death promoter (*bad*), Cytochrome-C (*cyt-c*), and *caspase3*, reached their highest values in August (*p* < 0.05). The expression levels of *bax* showed a trend of increasing and then decreasing from March to July (Figure 8A). The *bcl2* expression levels were constant from April to July, and then reached a maximum in August (*p* < 0.05) (Figure 8B). The *bid* expression levels did not differ from March to May, but gradually increased from June to August and reached a maximum in August (*p* < 0.05) (Figure 8C). The expression level of *caspase 3* was low from March to July, with no difference in April or May, and no difference in March, June, or July (*p* > 0.05) (Figure 8D). The protein expression level of *caspase 3* was similar to the gene expression level, where there was no difference in expression from March to July, and then reached a maximum in August (*p* < 0.05) (Figure 8K,L). The expression levels of caspase8 and caspase9 were both highly expressed in August (Figure 8G,H). The expression levels of PI3K and AKT did not differ much from month to month and showed a decreasing trend (Figure 8I,J).

## 4. Discussion

### 4.1. Delayed Sperm Discharge and Sperm Regeneration of Largemouth Bass during Out-of-Season Breeding

Experimental results demonstrated that sustained cold temperature stimulation caused delayed reproduction in largemouth bass. The histological results showed that there were two stages in the out-of-season breeding of LMB, the first being the delayed spermatogenesis activity of LMB. Normally, Sichuan LMB will be inseminated at the end of April [14]. However, in this study, the onset of sperm discharge in LMB was delayed to early June due to the low-temperature stress. The second stage comprises rapid maturation of sperm in July and August, suggesting that low-temperature stimulation of parental fish can initiate gonadal development. Similarly, Matthews [15] noted that sustained cold temperature (≤12 °C) stimulation is essential for gonadal development in counter-seasonal breeding. Our study suggests that sustained low temperatures are critical for delaying sperm expulsion and initiating secondary sperm development in off-season breeding in Sichuan LMB.

Gametogenesis and gonadal maturation in fish are regulated by the nervous and endocrine systems through the hypothalamus–pituitary–gonadal (HPG) axis. When external environmental factors change, hormone levels in fish are altered accordingly [16]. In the present study, the expression levels of relevant serum hormones were highest in April, which combined with the histological results suggest that during this period, the LMB exhibits ejaculatory behavior. In vertebrates, hormones are important physiological regulators. In the early stage of sperm development, the secretion of LH and FSH stimulates the maturation of spermatogenic tubules and promotes the secretion of T in vivo [17]. In addition, T can synergize with FSH to promote the initiation of spermatogenesis [18]. Furthermore, it has been demonstrated that testosterone and estradiol are closely associated with spermatogenesis [19,20]. Therefore, the levels of hormones are crucial for spermatogenesis and maturation. In this experiment, the high hormone levels in June may have been caused by the secondary development of the testes after sperm discharge in autumn-breeding LMB, and the gradual decrease in serum hormones in June, July, and August may be related to the gradual maturation of spermatozoa or to the decrease in hormone levels caused by low temperature [21].

In this experiment, the mRNA expression levels of hormone receptors also reached their highest values in April and May, thus suggesting the association with sperm discharge behavior of LMB. In Holland’s experiment, hormone receptors were all found to be highly expressed during the period of sperm discharge [22]. The elevated expression levels of *lhr* and *kiss2* may be related to the gradual maturation of the testes. The results of the current study indicate that exposure to temperatures of approximately 15 degrees Celsius can delay sperm discharge. This delay appears to be attributable to a retardation in developmental processes rather than to sperm degeneration. Conversely, excessive temperatures are known to cause sperm degeneration. The impact of lower temperatures on sperm development warrants further investigation. It is crucial to consider that while cold temperatures may influence sperm motility and lead to cessation of feeding in LMB, such effects could have broader implications for their overall health.

### 4.2. Histological Damage to Testis during Out-of-Season Breeding

Ultrastructural results of the spermatozoa indicated that during out-of-season breeding, sperm cells showed a separation of the plasma membrane in the head and swelling in the midsection. This result was similar to the plasma membrane damage in the head as well as swelling in the middle part caused by cryopreservation of spermatozoa [23]. Similar observations were reported in the marine pout (*Macrozoarces americanus*), in which thawed sperm showed varying degrees of swelling in the middle and head of the sperm and dehydration of the cell membrane [24]. The plasma membrane is the site of the most severe damage that occurs throughout low-temperature stress [25,26]. It has been suggested that the functional integrity of the sperm plasma membrane is critical in determining the fertility of individual spermatozoa [27]. Therefore, during the out-of-season breeding of LMB, damage to the plasma membrane of the sperm head due to cold stress will affect sperm quality. At the same time, aquaporin (AQP) is present in the sperm plasma membrane and mediates the passive transmembrane transport of free water molecules [28]. It has been demonstrated that AQP is involved in the spermatogenesis process and is associated with sperm low-temperature tolerance [29]. The results of the present study suggest that AQP begins to be highly expressed in June and that this may be associated with spermatogenesis. These alterations would result in a loss of mitochondrial function and a reduction in the energy reserves required for sperm flagellar motility, leading to abnormal physiological activity [30]. Plasma membrane integrity and mitochondrial function are critical determinants of successful egg fertilization. This study demonstrated that exposure to low temperatures adversely affects both plasma membrane integrity and mitochondrial function, resulting in a measurable decline in egg quality. Additionally, empirical observations from production environments reveal a reduced fertilization rate for largemouth bass during off-season periods compared to the normal breeding season. Consequently, future research should focus on developing strategies to mitigate the detrimental effects of low temperatures on sperm quality or enhancing pre-fertilization sperm viability. Such advancements could facilitate the successful off-season breeding of LMB.

### 4.3. Apoptosis in Spermatozoa during Out-of-Season Breeding

Apoptosis, also known as programmed cell death, is an important regulatory pathway for the body to maintain the stability of the internal environment in response to various extracellular and intracellular signals [31]. Previous studies have demonstrated that Bax, a pro-apoptotic protein that is involved in an intrinsic apoptotic pathway initiated by mitochondrial changes, and Bcl-2, an inhibitory apoptotic protein, are markers of apoptosis and are proteins that play critical roles in maintaining cell survival [32]. Activation of cysteinyl aspartate-specific proteases (caspases) triggers a cascade of reactions that ultimately lead to apoptosis, with caspases 8 and 9 acting as the primary initiators and caspase 3 as the primary executor [33]. Both biotic and abiotic factors can trigger apoptosis [34]. It has been experimentally demonstrated that the mRNA expression levels of apoptosis-related genes (*bax*, *bcl2*, and *caspase3*) increased in Nile tilapia under low-temperature stress [35]. The results of this study also showed that the expression levels of *bax*, *bcl2*, and *caspase3* in LMB increased after low-temperature stress. This indicated that spermatozoa of LMB underwent apoptosis during the autumn reproduction process. The mRNA expression levels of BH3-interaction domain death agonist (BID) and cytochrome C (CYT-C), important pro-apoptotic proteins of the mitochondrial apoptotic pathway [36], showed increasing trends during this period, indicating that the apoptosis of LMB during out-of-season breeding could be achieved through the mitochondrial apoptotic pathway.

Spermatid apoptosis, which is regulated by multiple signaling pathways and various internal and external factors, participates in the entire process of spermatogenesis and maturation [37,38]. In this study, the expression levels of apoptosis-related genes in the testes of LMB were significantly increased in the final month. This may have been caused by various factors such as the level of testosterone in the testes, different stages of sperm development, and the degree of oxidative stress in the body. At the same time, the significant increase in the expression levels of apoptotic genes in the testes in the final month may be one of the reasons for the low sperm quality in the off-season reproduction of LMB.

### 4.4. Oxidative Stress in the Testis during Out-of-Season Breeding

Changes in temperature can increase the rate of formation of endogenous reactive oxygen species (ROS) in aquatic organisms, leading to excessive accumulation of ROS in vivo [39]. The excessive production of ROS in cells leads to cellular lipid peroxidation, DNA damage, protein denaturation, and even programmed cell death [40]. Low-temperature stress causes fish to be in oxidative stress, resulting in an increase in the concentration of reactive oxygen radicals that disrupts the dynamic balance of free radical production and elimination [41]. Fish can eliminate excess free radicals such as superoxide anions, hydroxyl radicals, and hydrogen peroxide by producing more antioxidant enzymes (e.g., SOD, CAT, and GPX) to alleviate the effects of oxidative stress [42]. SOD is one of the most important antioxidant enzymes that can convert intracellular oxygen radicals (O_2_^−^) into hydrogen peroxide (H_2_O_2_) and molecular oxygen (O_2_) [43]. While CAT plays a major role in neutralizing H_2_O_2_ by converting it to H_2_O and O_2_, GPx is involved in detoxifying H_2_O_2_ and organic hydrogen peroxide [44]. In this study, with the increase in the duration of low-temperature stress, the mRNA expression levels of the antioxidant genes *sod* and *gpx* in the testis showed increasing trends, indicating that the LMB suffered from oxidative stress under low-temperature stress, and that this stimulated the activity of relevant antioxidant enzymes. The mRNA expression levels of cat showed an initial decrease, followed by an increase. The protein expression levels of cat showed the same trend. The reason for this phenomenon may be that the production of free radicals in the organism exceeds the scavenging ability, leading to oxidative damage and resulting in a decrease in expression levels [45].

The ECH-related protein 1 (Keap1)-Nrf2 signaling pathway, an important antioxidant pathway in vivo, is activated when the organism is subjected to oxidative stress [46]. Under normal physiological conditions, Keap1 in the cytoplasm binds to Nrf2 and progressively degrades Nrf2. Upon oxidative stress, Keap1 dissociates from Nrf2 and enters the nucleus and binds to intracellular antioxidant response elements, thus causing transcription of a series of downstream antioxidant enzymes and finally altering the antioxidant capacity of the organism [47]. In the present study, the mRNA expression levels of both *keap1* and *nrf2* were increased, suggesting that LMB activates the nrf2-keap1 defense system to participate in the regulation of oxidative stress under prolonged low-temperature stress. Hsp70 is a ubiquitous molecular chaperone, an important regulatory as well as protein repair element of the organism and is thought to be important in protecting cells from oxidative stress as well as having anti-apoptotic effects [48]. In this experiment, the mRNA level expression of *hsp70* in the testis gradually increased, indicating that HSP70 could enhance the tolerance of the organism to low-temperature stress. These results indicate that prolonged exposure to low-temperature stress induces oxidative stress in the sperm of largemouth bass (LMB), subsequently triggering a sustained activation of the antioxidant system. Additionally, it is observed that spermatogenesis events occurring in June are associated with a reduction in spermatogenic antioxidants and an attenuation of the antioxidant system. This suggests that a period following spermatogenesis represents a vulnerable phase for the fish, necessitating more meticulous management to support their overall health and reproductive efficiency.

## 5. Conclusions

This study elucidates that low-temperature stress can impede sperm development and delay spermatogenesis in largemouth bass. Exposure to low temperatures negatively impacts sperm by affecting both its structural integrity and functional capacity, thereby reducing the fertilization rate. Notably, the development of largemouth bass sperm may be completed in as little as two months or less. The roles of antioxidant defense and apoptosis are pivotal in preserving sperm quality under such stress conditions. This research contributes new insights into the molecular mechanisms that influence sperm quality during off-season reproduction in fish. It offers valuable insights for devising future strategies to protect sperm, potentially guiding the development of effective preservation and enhancement methods under stressful conditions.

## Data Availability

Data will be made available on request.

## Supplementary Materials

The following supporting information can be downloaded at: https://www.mdpi.com/article/10.3390/antiox13091077/s1. Supplementary Figure S1. The temperature of the water sampled monthly. Supplementary Figure S2. The anatomy of the testes tissues in each month. Supplementary Figure S3. Electron micrographs of largemouth black bass May sperm (scale bar: 500 nm). Supplementary Table S1. Primer sequences for real-time PCR.
