# Supplementary material for: Delayed Reproduction, Injury, and Regeneration of Testes in Out-of-Season Breeding of Largemouth Bass (Micropterus nigricans)
Source: Antioxidants (Basel). 2024 Sep 4;13(9):1077. doi: 10.3390/antiox13091077 (PMC11429289; doi:10.3390/antiox13091077)
Supplement: Supplementary file 1 [file antioxidants-13-01077-s001.zip › antioxidants-3111198-supplementary.pdf]

Supplementary Figure S1. The temperature of the water sampled monthly

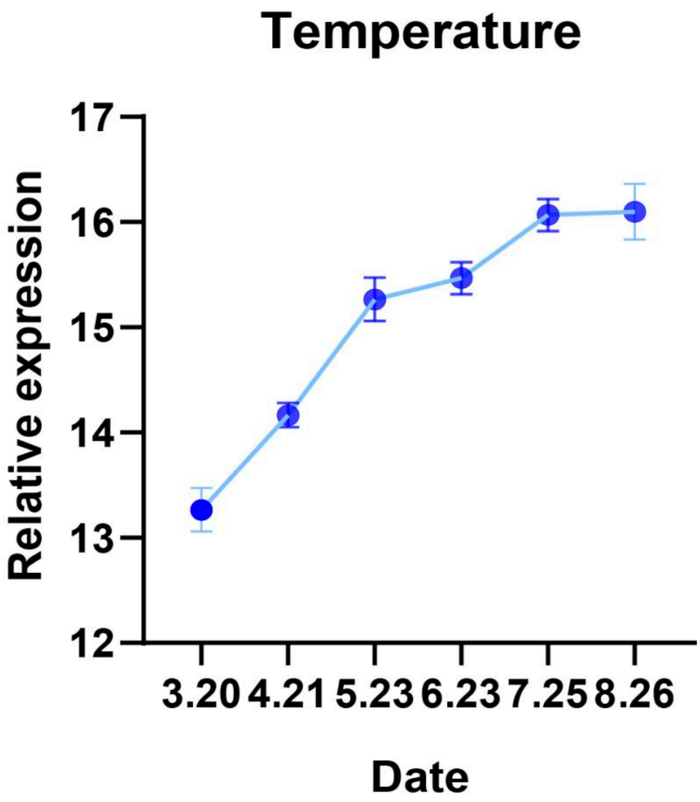

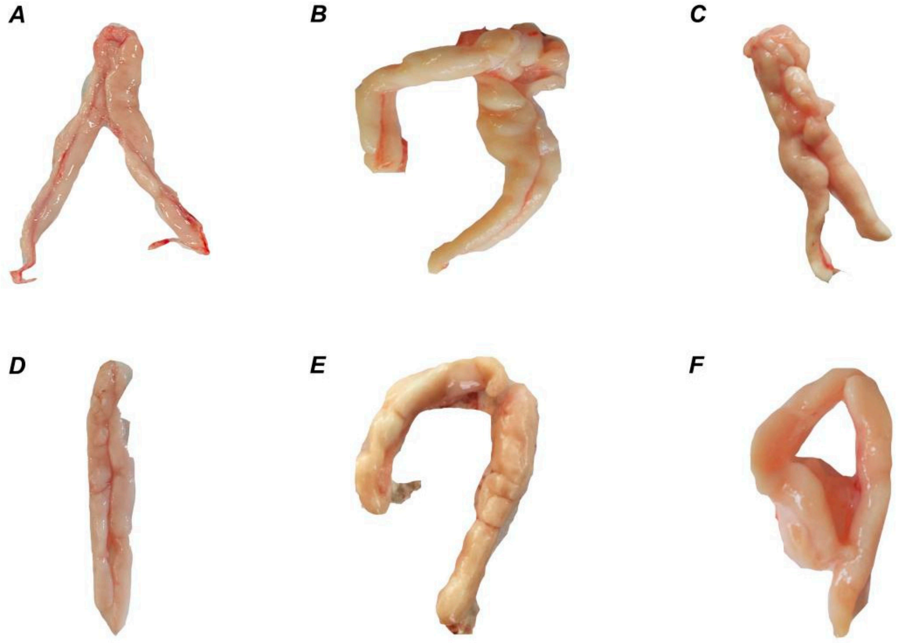

Supplementary Figure S2. The anatomy of the testes tissues in each month. A-F corresponds to the anatomy of the testes tissues in March, April, May, June, July, and August, respectively.

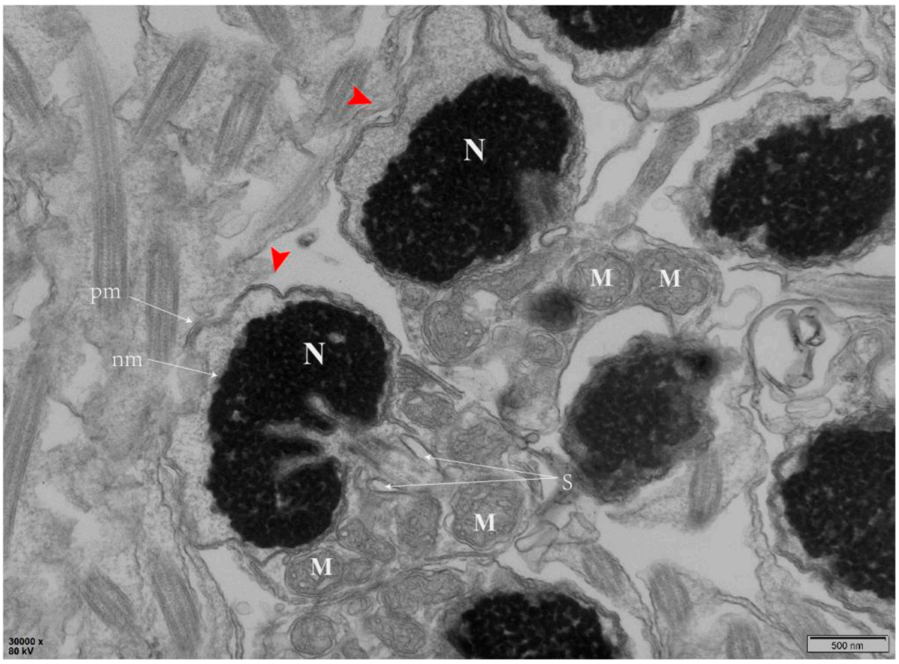

Supplementary Figure S3. Electron micrographs of largemouth black bass May sperm (scale bar: 500 nm). N: nucleus; M: mitochondria; red arrow: head plasma membrane separation.

Supplementary Table S1. Primer sequences for real-time PCR.

Gene	Primers	Sequence5'-3'	Target size(bp)	TM（℃）	Accession number
18S	F	CGGCTACCACATCCAAGGAA	86	59.5	XM020154709.1
	R	CCTGTATTGTTATTTTTCGTCACTACCT			
β-actin	F	AAAGGGAAATCGTGCGTGAC	163	61.0	XM020651307.1
	R	AAGGAAGGCTGGAAGAGGG			
CAT	F	GTTCCCGTCCTTCATCCACT	85	60.4	MK614708.1
	R	CAGGCTCCAGAAGTCCCACA			
SOD1	F	CCCCACAACAAGAATCATGC	178	58	MK614709.1
	R	TCTCAGCCTTCTCGTGGA			
Caspase3	F	GCTTCATTCGTCTGTGTTC	98	54.0	ASM2243578v1
	R	CGAAAAAGTGATGTGAGGTA			
Bax	F	ACTTTGGATTACCTGCGGGA	133	61.0	ASM2243578v1
	R	TGCCAGAAATCAGGAGCAGA			
Bcl2	F	CGCCATCCACAGAGTCCT	145	59.4	ASM2243578v1
	R	CCGGAACAGTTCGTCTATCACC			
Keap1	F	CAGCATTACATGGCCGCATC	86	55.0	ASM2243578v1
	R	CTTCTCTGGGTCGTAAGACTCC			
Nrf2	F	CAGACAGTTCCTTTGCAGGC	116	56.9	ASM2243578v1
	R	AGGGACAAAAGCTCCATCCA			
Lhr	F	AGTCTACACCCTTTCCACC	285	64.0	ASM2243578v1
	R	AGCACATAGCAAACGCAC			
Star	F	CAGAGAGCAACGGGGAT	114	54.7	ASM2243578v1
	R	CAAACAGGAGGTCATAGAGT			
Fshr	F	GTGAGTTGGTGGTGCTTG	204	57.9	ASM2243578v1
	R	ATCTGTTCCTGTGTATGTTTTT			

Cyp17a1	F	TTTTTGCCGTGCTGCT	179	64.0	ASM2243578v1
	R	CGTCTGTCCGTATTTCTCC			
Er1	F	GGAAACCACCTCAACACC	210	61.3	ASM2243578v1
	R	CAGGACCACACCCCGTA			
AQP-1	F	CGTCTGACCGTGAGTGA	179	62.0	ASM2243578v1
	R	AGGTTGTTTGGGTGGAT			
AQP-3	F	TGGTGTTGAGTGGAGGTT	118	62.0	ASM2243578v1
	R	GCAGGGTTCAGATGGC			
hsp70	F	ACGCAAAGAGGGAATGAG	174	53.0	ASM2243578v1
	R	AGATACTGGGAGAAGCACAA			
kiss2	F	GCTGATTGTTGGTCACGGAG	148	64.8	ASM2243578v1
	R	CAGGGAGAAGCACAGGTTTG			
cirbp	F	AGATGGGCGGGTTATTC	133	64.8	ASM2243578v1
	R	TCCCCTGTCTCCGTTGT			
bid	F	GGAGGTGGACAGGGTGA	131	64.8	ASM2243578v1
	R	AGAGGTTTCGCAGCAGTT			
ar	F	ATGGATGGGGGTGATGGT	296	57.9	ASM2243578v1
	R	CGAGTTCGTTGATGTAGGTAA			

F: forward primer; R: reverse primer.
